# 

**Published:** 1852-12

**Authors:** 


*American Medical Association.*—The Sixth Annual Meeting of this Association will be held at the city of New York, on Tuesday, May 3d, 1853.

The secretaries of all societies, and other bodies entitled to representation in this Association, are requested to forward to the undersigned correct lists of their respective delegations as soon as they may be appointed; and it is desired by the Committee of Arrangements that these appointments be made at as early a period as possible.

The following is an extract from Article II of the Constitution:

"Each local society shall have the privilege of sending to the Association one delegate for every ten of its regular resident members, and one for each additional fraction of more than half this number. The faculty of every regularly constituted medical college or chartered school of medicine, shall have the privilege of sending two delegates. The professional staff of every chartered or municipal hospital containing a hundred inmates or more, shall have the privilege of sending two delegates; and every other permanently organized medical institution of good standing, shall have the privilege of sending one delegate.

EDWARD L. BEADLE,

*One of the Secretaries of Am. Med. Association.*

42 Bleecker street, New York.

The medical press of the United States is respectfully requested to copy the above.

